# Effective energy storage from a triboelectric nanogenerator

**DOI:** 10.1038/ncomms10987

**Published:** 2016-03-11

**Authors:** Yunlong Zi, Jie Wang, Sihong Wang, Shengming Li, Zhen Wen, Hengyu Guo, Zhong Lin Wang

**Affiliations:** 1 School of Materials Science and Engineering, Georgia Institute of Technology, Atlanta, Georgia 30332, USA; 2 Beijing Institute of Nanoenergy and Nanosystems, Chinese Academy of Sciences, Beijing 100083, China

## Abstract

To sustainably power electronics by harvesting mechanical energy using nanogenerators, energy storage is essential to supply a regulated and stable electric output, which is traditionally realized by a direct connection between the two components through a rectifier. However, this may lead to low energy-storage efficiency. Here, we rationally design a charging cycle to maximize energy-storage efficiency by modulating the charge flow in the system, which is demonstrated on a triboelectric nanogenerator by adding a motion-triggered switch. Both theoretical and experimental comparisons show that the designed charging cycle can enhance the charging rate, improve the maximum energy-storage efficiency by up to 50% and promote the saturation voltage by at least a factor of two. This represents a progress to effectively store the energy harvested by nanogenerators with the aim to utilize ambient mechanical energy to drive portable/wearable/implantable electronics.

## Abstract (web summary)

Self-charging systems based on the connection of a nanogenerator and an energy storage unit through a rectifier can have low energy storage efficiencies. Here, the authors design the charging cycle to maximize the energy storage efficiency of a triboelectric nanogenerator by introducing a motion-induced switch.

## Graphical abstract

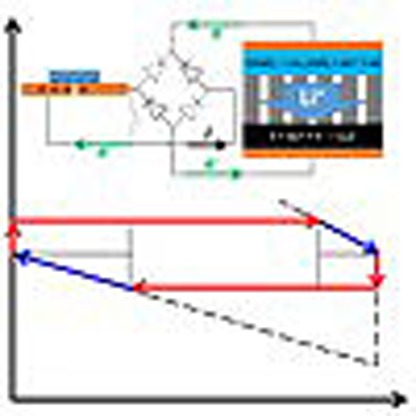

With the rapid development of mobile and portable electronics, more and more efforts have been dedicated to looking for sustainable mobile energy source at the power levels of micro-to-milli watts for these electronics. Currently, the major approach for powering these electronics is by using energy storage units such as batteries^1^,^2^,^3^ and capacitors^4^,^5^. However, the main drawback of these energy storage units is the limited lifetime, so that they cannot drive the electronics sustainably. Alternatively, to drive small electronics sustainably through harvesting ubiquitous ambient small-scale energy, nanogenerators^6^ based on piezoelectric, pyroelectric and triboelectric effects have been developed, which are dramatically different from traditional generators. Among nanogenerators, triboelectric nanogenerators (TENG)^7^,^8^,^9^,^10^ have attracted attention due to their high output and high energy conversion efficiency. Hence, our study here mainly focuses on TENGs. The fundamental working mechanism of a TENG is based on coupling of triboelectrification and electrostatic induction^10^,^11^,^12^. First, at least one pair of triboelectric layers made from materials with distinct electron affinities get into physical contact to create triboelectric charges. Second, as triggered by external mechanical force, the relative motion between the triboelectric layers breaks the balanced electrostatic charge distribution on electrodes. As a result, the potential difference between electrodes is built and free electrons flow through external circuits to achieve new equilibrium. When the triboelectric layers move back, the free electrons flow back to return to the original electrostatic equilibrium. Under periodical mechanical motions such as vibrations^13^,^14^,^15^, human walking^16^,^17^ and ocean waves^18^,^19^, pulsed alternating current (AC) output is delivered via the TENG to external circuit. Due to the nature of variable frequency and irregular amplitude of the pulsed AC output, TENGs cannot be directly used to drive most electronic devices. An energy storage unit is required to store the energy harvested by nanogenerators and to provide a regulated and manageable output. Consequently, self-charging power systems^20^,^21^,^22^ have been developed by hybridizing a nanogenerator and an energy storage unit, and the latter is charged by the former through a full-wave bridge rectifier.

However, most of the previous research on TENGs mainly focused on output performance under external load resistances^12^,^17^,^23^,^24^,^25^,^26^; while only a few papers^20^,^22^,^27^,^28^ explored the process of using a TENG to charge an energy storage unit, from which we obtained initial understandings on the charging characteristics. From these studies, it has been demonstrated that the charging rate decays quickly after several charging cycles. The saturation voltage, which is the highest achievable voltage of the energy storage unit, is much smaller than the open-circuit voltage of the TENG, resulting in a low energy-storage efficiency regardless of the energy conversion efficiency of the TENG. Therefore, studies are required to further understand the charging process and to improve the energy storage performances for TENGs. According to a recent study on the standards of TENGs, the average output power is mainly determined by the encircled area of its operation cycle in the built-up voltage *V* – transferred charge *Q* plot^12^. Therefore, the corresponding operation cycle for charging the energy storage units should be studied by considering the charging process of the TENG to achieve the maximized energy-storage efficiency.

In this work, we first analysed the operation cycle of using a TENG to directly charge a battery/capacitor through a bridge rectifier by our recently proposed *V*–*Q* plot^12^. A sliding freestanding-triboelectric-layer (SFT) mode TENG was fabricated to experimentally measure the *V*–*Q* plots of the direct charging cycle. Then a rationally designed cycle was proposed to improve the charging rate and the saturation voltage. The voltage of the energy storage unit was demonstrated to be a key parameter for optimizing the stored energy per cycle. As a proof of concept, a motion-triggered switch was added to the fabricated TENG to achieve this designed charging cycle. Furthermore, the enhanced performances including the enhanced charging rate, the improved energy-storage efficiency and the promoted saturation voltage were achieved experimentally. Finally, the self-charging power system operated under this designed charging cycle was used to drive a commercial calculator in the sustainable mode. Our work represents a paradigm shift in strategies and experimental methods to achieve effective energy harvesting and storage by TENGs as well as other nanogenerators.

## Results

### Theoretical analysis of the direct charging cycle

**Figure 1:**
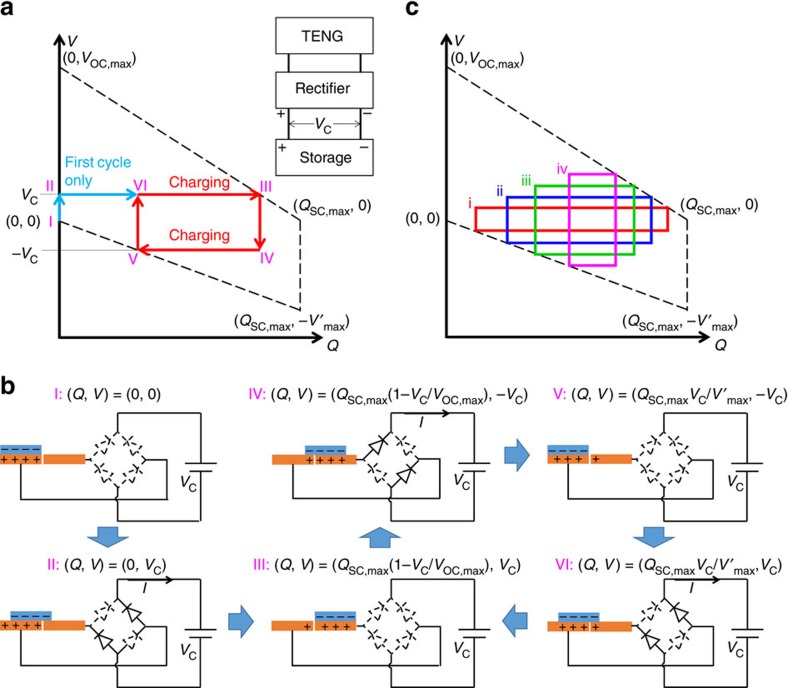
The *V*–*Q* plot and the physical process of the direct charging cycle. (**a**) The *V-Q* plot. The inset of **a** shows the circuit diagram of the system. The dashed lines in **a** represent the cycle with the maximized energy output with infinite resistance. (**b**) The schematic diagrams of the physical process. In **b**, the diodes drawn by solid and dashed lines are for the on and off states, respectively. (**c**) The direct charging cycles (the second cycle and thereafter) for different charging voltages (voltage iv>iii>ii>i).

Conventional integration of a TENG and an energy storage device was achieved through a full-wave bridge rectifier, as shown in the inset of Fig. 1a. We utilize the *V*–*Q* plot as the analytical tool for TENG energy storage, as shown in Fig. 1, in which *V* is the potential difference between the two electrodes, and *Q* is the amount of charge transferred between the electrodes. Several important parameters of the TENG are defined in Table 1. The parameters for TENG energy storage are given in Supplementary Table 1. We use the most commonly utilized minimum achievable charge reference state^29^, so both *Q* and *V* at *x*=0 position are set to be 0 initially, where *x* is the relative displacement between triboelectric layers, which is used to specify the operation of the TENG. The cycle for maximized energy output with infinite load resistance, which has the largest possible energy output per cycle *E*_m_, is also plotted in Fig. 1a,c using the dashed lines (with equations given in Supplementary Note 1)^12^. Here we assume that the charging voltage *V*_C_, which is the voltage of the energy storage unit, does not change significantly during one cycle of the TENG operation (as stated in Supplementary Note 2).

Therefore, starting from (*Q*, *V*)=(0, 0), the direct charging cycles of the TENG can be described with the following steps (Fig. 1 a,b). For the first cycle, the first step is to change from status I to II. The initial status I of the system is at (*Q*, *V*)=(0, 0) and *x*=0. Because the initial voltage *V* supplied by the TENG is lower than the charging voltage *V*_C_, all of the diodes in the rectifier are at ‘off' state, which makes the external circuit condition close to the open-circuit condition. When the operation of the TENG starts, *V* begins to increase with *x*. No charge transfer occurs during this step, until *V* reaches *V*_C_ as shown in status II.

The second step is to change from status II to III. Two of the four diodes in the rectifier are turned on to enable the charging process. Then *x* continues to increase until *x*=*x*_max_, during which the TENG charges the battery/capacitor at *V*=*V*_C_ (See Supplementary Note 3 and Supplementary Fig. 1 for the detailed process) until status III is achieved. Please note that in status III the charges cannot be fully transferred to the other electrode (*Q*<*Q*_SC,max_), since *V*=*V*_C_ should be maintained during the charging process in this step.

The third step is to change from status III to IV: *x* starts to decrease. Since the reversed-direction current is forbidden, *V* decreases without charge transfers, until *V*=−*V*_C_ as shown in status IV. During this step all of the diodes in the rectifier are off because the net voltage |*V*| is lower than the threshold voltage at *V*_C_.

The fourth step is to change from status IV to V. The other two diodes in the rectifier are turned on to enable the charging process again. *x* continues to decrease until *x*=0, as shown in status V, during which, the TENG charges the battery/capacitor with *V*=−*V*_C_. Similar to status III, in status V the charge cannot be fully transferred back to the original electrode (*Q*>0), since *V*=−*V*_C_ should be maintained during the charging process in this step. Here the first charging cycle is completed.

For the second cycle and thereafter (starting from status V), *x* starts to increase, all of the diodes in the rectifier are off (since |*V*|<*V*_C_) and *V* increases without charge transfers, until *V*=*V*_C_ as shown in status VI. *x* continues to increase and the corresponding two diodes are turned on to allow the charging process continue until *x*=*x*_max_, the same as status III. The process to complete one cycle is the same as steps 3 and 4 of the first cycle, which comes back to status V. Therefore, the second cycle and thereafter follow the sequence of V→VI→III→IV→V, which is the steady-state charging cycle.

The corresponding *V*–*Q* cycle is plotted in Fig. 1a, with the (*Q*, *V*) coordinates corresponding to statuses calculated in Supplementary Note 4. The stored energy per cycle *E*_C,direct_, which is proportional to the average output power, can be calculated as the encircled area of each cycle^12^:


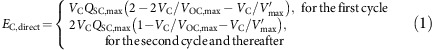


Since the charging of an energy storage device requires many cycles of operation of the TENG, we will only consider the equation for the steady-state cycles. The amount of charge (*Q*_C,direct_) flowing into the energy storage unit per cycle can be calculated as *E*_C,direct_/*V*_C_, which equals to the total length of the sides that are parallel to the *Q* axis in the *V–Q* plot. The direct charging cycles corresponding to different charging voltages *V*_C_ are plotted in Fig. 1c, which shows the decrease of *Q*_C,direct_ due to the increase of *V*_C_ (which is further discussed in Supplementary Note 5).

From equation (1), with varied *V*_C_, we can derive that the maximum value of *E*_C,direct_ is achieved when 
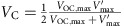
, which is:


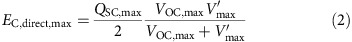


We define the energy storage efficiency *η* as the percentage of the stored energy per cycle in the largest possible energy output per cycle *E*_m_. Therefore, for the direct charging cycle, the maximum energy-storage efficiency is:


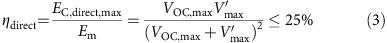


And the largest possible *V*_C_ to operate the direct charging cycle is at the saturation voltage:


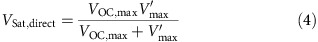


### Experimental demonstration of the direct charging cycle

**Figure 2:**
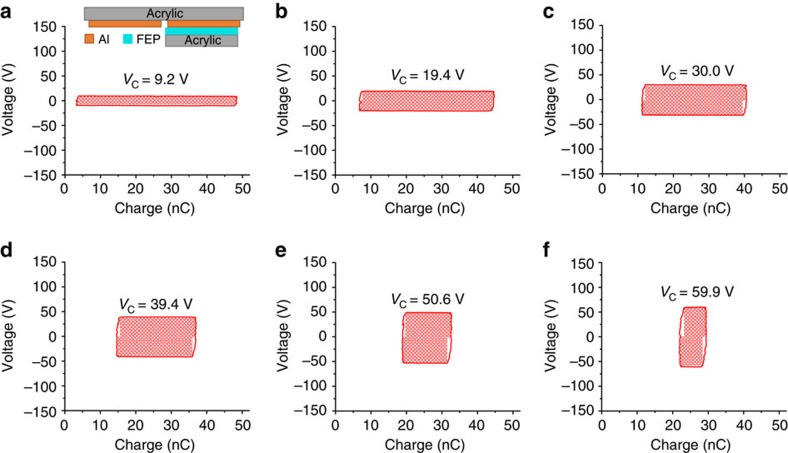
The *V*–*Q* plots of the direct charging cycle for a commercial capacitor with charging voltage *V*_C_ increased from 9.2 V to 59.9 V. For these plots, *V*_C_ are (**a**) 9.2 V, (**b**) 19.4 V, (**c**) 30.0 V, (**d**) 39.4 V, (**e**) 50.6 V and (**f**) 59.9 V. The inset of **a** shows the TENG structure. The shaded areas are the stored energy per cycle *E*_C,direct_.

A SFT mode TENG is fabricated to measure the direct charging cycle experimentally. The TENG is schematically illustrated in the inset of Fig. 2a. The top static part of the TENG is made by attaching two parallel aluminium (Al) electrodes on the bottom surface of an acrylic board. The moving part of the TENG is made by attaching a piece of fluorinated ethylene propylene (FEP) film on the top surface of another acrylic board. The FEP film and Al electrodes are the triboelectric layers. The fabrication process is described in detail in the Methods section. In this structure, *V*_OC,max_=*V*′_max_ since the capacitance between the two electrodes is kept identical^29^,^30^. The measured cycle for maximized energy output with infinite load resistance is shown in Supplementary Fig. 2b, where *V*_OC,max_=*V*′_max_≈140 V, and *Q*_SC,max_≈50 nC. The circuit diagram for this measurement is shown in Supplementary Fig. 2a. A commercial capacitor (0.73 μF) and fabricated lithium-ion batteries connected in series are used as the energy storage devices, respectively. For the capacitor, the charging voltage *V*_C_ increases gradually during the charging process, and the *V*–*Q* curves of direct charging cycle with different *V*_C_ are plotted in Fig. 2. For the batteries, each battery can supply a voltage of about 3–4 V, and different number of batteries are connected to achieve different *V*_C_. The fabrication method of the batteries and the measurement set-up are described in the Methods section and Supplementary Fig. 3, respectively. The *V*–*Q* curves of the direct charging cycle for batteries are plotted in Supplementary Fig. 4. From these *V*–*Q* curves, we notice that all of the curves in the direct charging cycle for both batteries and the capacitor have a rectangular shape. The total length of the two sides parallel to the *Q* axis can be directly used as *Q*_C,direct_, as demonstrated in Supplementary Note 6 and Supplementary Fig. 5. *Q*_C,direct_ decreases with increasing *V*_C_, and the saturation voltage *V*_Sat,direct_ is measured at almost 70 V, which are consistent with the calculated results from equations (1) and (4).

### Theoretical analysis of the designed charging cycle

**Figure 3:**
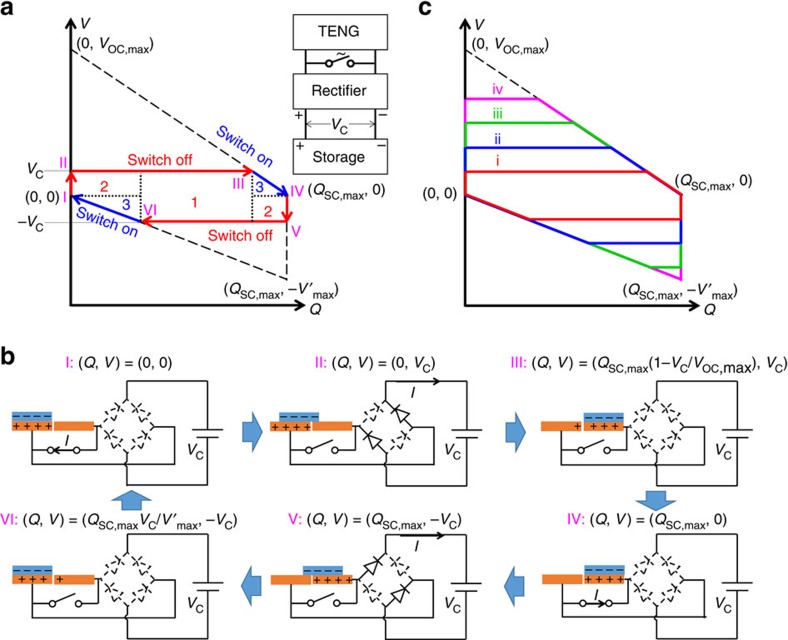
The *V*–*Q* plot and the physical process of the rationally designed charging cycle. (**a**) The *V-Q* plot. The inset of **a** shows the circuit for the designed charging cycle. The dashed lines in **a** represent the cycle of maximized energy output with infinite resistance. (**b**) The schematic diagrams of the physical process. In **b**, the diodes drawn by solid and dashed lines are for the on and off states, respectively. (**c**) The designed charging cycle for different charging voltages, with charging voltage intensity iv>iii>ii>i.

From the equations and experimental results presented above, we can make three observations on the direct charging cycle. First, the charge flowing into the energy storage unit per cycle *Q*_C,direct_, which is proportional to the charging rate, quickly decreases with the increased *V*_C_. Second, the maximum value of the energy storage efficiency *η* is only 25%. Finally, the saturation voltage *V*_Sat,direct_ is much smaller than *V*_OC,max_ and *V*′_max_. To improve these charging performances, we rationally design a charging cycle. A switch is added in parallel to the TENG, which is controlled to instantaneously turn on only at *x*=0 and *x*=*x*_max_, and turn off at all the other *x*. Starting from (*Q*, *V*)=(0, 0), the process of the designed charging cycle can be described as follows (Fig. 3a,b):

Steps 1 and 2 (status I–III) are the same as those in the first cycle of the direct charging cycle. Similarly, in status III the charge cannot be fully transferred to the other electrode (*Q*<*Q*_SC,max_).

Step 3 (status III–IV): while in status III, *x* reaches *x*_max_, and then the switch is turned on. The charge transfers in short-circuit condition to achieve (*Q*, *V*)=(*Q*_SC,max_, 0), which is status IV.

Step 4 (status IV–V): the switch is turned off as *x* begins to decrease; at the same time all of the diodes in the rectifier are off since |*V*|<*V*_C_. Therefore, *V* decreases without any charge transfers until it reaches −*V*_C_, which is status V.

Step 5 (status V–VI): the second half-cycle of the charging starts as *x* continues decreasing, until *x*=0 at status VI. Also, in status VI the charge cannot be fully transferred back to the original electrode (*Q*>0).

Step 6 (status VI to I): the switch is turned on again to make (*Q*, *V*) return to (0, 0), that is, status I.

Therefore, the repeated process for the designed charging cycle is through I→II→III→IV→V→VI→I. As concluded from steps 3 and 6, the function of the switch is to enable the complete charge transfer by creating an instantaneous short-circuit condition. Consequently, in the next half-cycle, there are more charges available to charge the energy storage unit, which is the key to enhance the charging performances by using the designed charging cycle.

By marking the coordinates of corresponding statuses (as calculated in Supplementary Note 4) in the *V*–*Q* plot (Fig. 3a), we observe that there are three regions of energy in the encircled area in the designed charging cycle. Area 1 represents the energy that can be stored in both the direct and the designed charging cycles; area 3 represents the energy released through the switch; and the energy of area 2 is the part that can only be stored in the designed charging cycle. Thus, the stored energy per cycle in the designed charging cycle should only include energies in areas 1 and 2. During steps 1–3 and steps 4–6, the energies stored are derived as 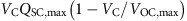 and 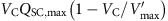, respectively. Therefore, the total stored energy per cycle is:


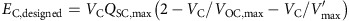


Note that this equation is only valid for *V*_C_≤min {*V*_OC,max_, *V*′_max_}. Considering the symmetric roles of *V*_OC,max_ and *V*′_max_ (as explained in Supplementary Note 7), we can simply assume *V*_OC,max_≥*V*′_max_. If *V*′_max_<*V*_C_≤*V*_OC,max_, since during step 4 the voltage cannot achieve –*V*_C_, there is no energy stored during steps 4–6. Then the equation becomes:


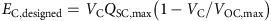


Therefore:


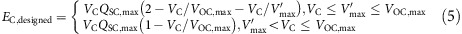


Similarly, the other characteristic parameters of the designed charging cycle can be calculated as follows:

The maximum stored energy per cycle (achieved at 
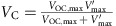
 or 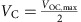):


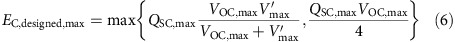


The maximum energy storage efficiency:


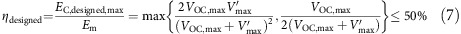


And the saturation voltage is:


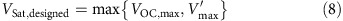


As derived by these equations of the designed charging cycle: the amount of charge flowing to the battery/capacitor per cycle of the designed charging cycle (*Q*_C,designed_) is larger than that of the direct charging cycle (*Q*_C,direct_), corresponding to the same *V*_C_ (Supplementary Note 5); the maximum energy storage efficiency has been increased up to 50%; and the saturation voltage *V*_Sat,designed_ is the same as the larger one in *V*_OC,max_ and *V*′_max_, which is at least twice the value of *V*_Sat,direct_. Therefore, this designed cycle represents an effective strategy to greatly enhance the performances of using TENG to charge an energy storage unit.

### Experimental demonstration of the designed charging cycle

**Figure 4:**
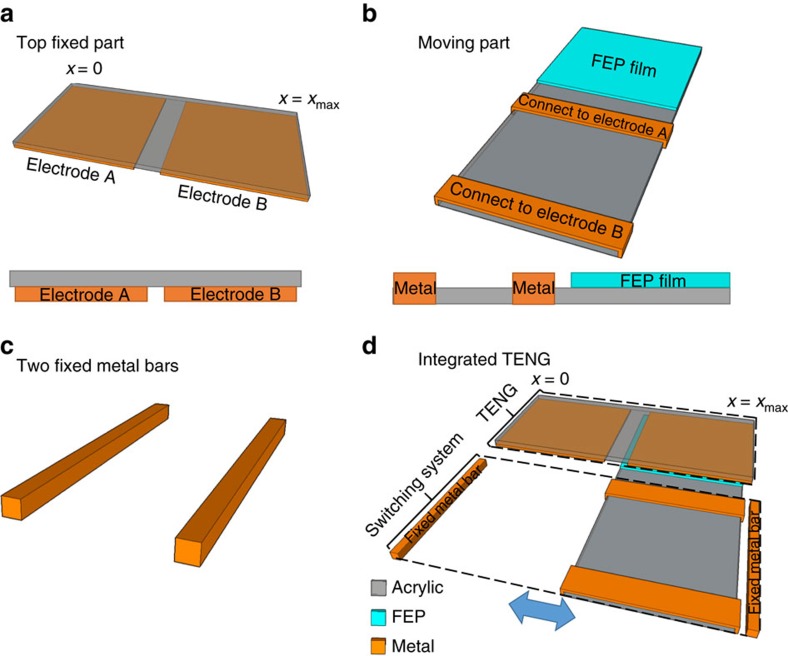
The TENG with the motion-triggered switch fabricated for the designed charging cycle. This TENG is composed of **a** the static part, (**b**) the moving part and (**c**) the two fixed metal bars. (**d**) The TENG as integrated by the three parts in **a**–**c**. The insets in **a**,**b** show the front view of the static part and the right-side view of the moving part.

We demonstrate this designed charging cycle experimentally by adding a motion-triggered switch on the fabricated SFT mode TENG, which is similar to the design of the pulsed nanogenerator as reported previously^31^, as shown in Fig. 4. The top static part is kept the same (Fig. 4a). Two parallel titanium (Ti) ribbons are attached on the acrylic board of the moving part (Fig. 4b). These two Ti ribbons are connected to the two Al electrodes through copper wires, respectively. Two Ti bars (Fig. 4c) are fixed in the positions of *x*=0 and *x*=*x*_max_. The configuration of the integrated structure is shown in Fig. 4d and the detailed fabrication process is described in the Methods section. When *x*=0 or *x*=*x*_max_, both of the two Ti ribbons contact with one of the Ti bars, and then the two electrodes are connected, which represents the switch on. Otherwise, the electrodes are not connected, which represents the switch off.

**Figure 5:**
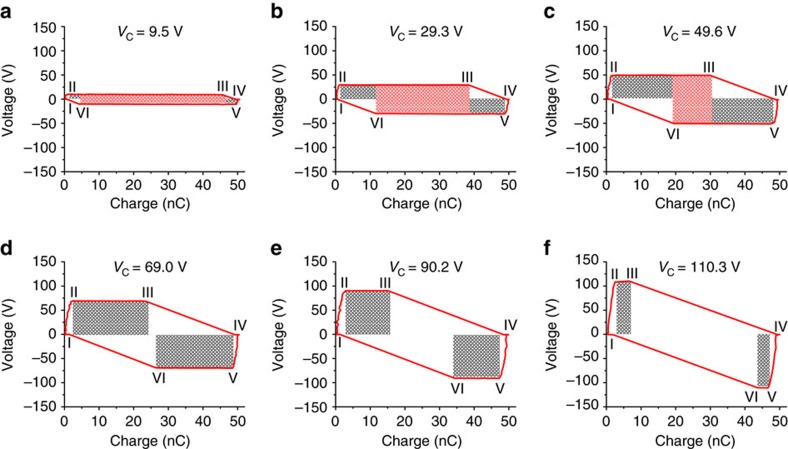
The *V*–*Q* plots of the designed charging cycle for a commercial capacitor with charging voltage increased from 9.5 V to 110.3 V. For these plots, *V*_C_ are (**a**) 9.5 V, (**b**) 29.3 V, (**c**) 49.6 V, (**d**) 69.0 V, (**e**) 90.2 V and (**f**) 110.3 V. The six statuses in the designed charging cycle are marked in the plots. The shaded areas are the stored energy per cycle by the designed charging cycle *E*_C,designed_, in which the red shaded areas are the part of the energy that can also be stored in the direct charging cycle with the same *V*_C_, while the grey shaded areas are the ‘extra' energy that can only be stored in the designed charging cycle.

The *V*–*Q* curves of these TENGs operated in the designed charging cycle are measured. Figure 5 presents the *V*–*Q* plots of the designed charging cycle for the same capacitor used in the experiments for the direct charging cycle, and the plots for the batteries in series are shown in Supplementary Fig. 6. Similarly, the total length of the two sides that are parallel to *Q* axis in *V–Q* plot can be used directly as *Q*_C,designed_. The stored energy per cycle *E*_C,designed_ is marked as the shaded area in all the plots in Fig. 5, in which the red and grey shaded areas represent areas 1 and 2 in Fig. 3a, respectively. Compared with the direct charging cycle, the designed charging cycle can store more energy per cycle for the same *V*_C_, and *V*_Sat_ can be enhanced to be close to 140 V. To further understand the *V*–*Q* plots in Fig. 5d,f when *V*_C_ is larger than *V*_Sat,direct_ but smaller than *V*_Sat,designed_, the corresponding charging cycles are discussed in Supplementary Note 8, and the theoretically derived *V*–*Q* plots are displayed in Supplementary Fig. 7. All of these experimental results are consistent with our theoretical derivations.

### Quantitative comparisons between the charging cycles

**Figure 6:**
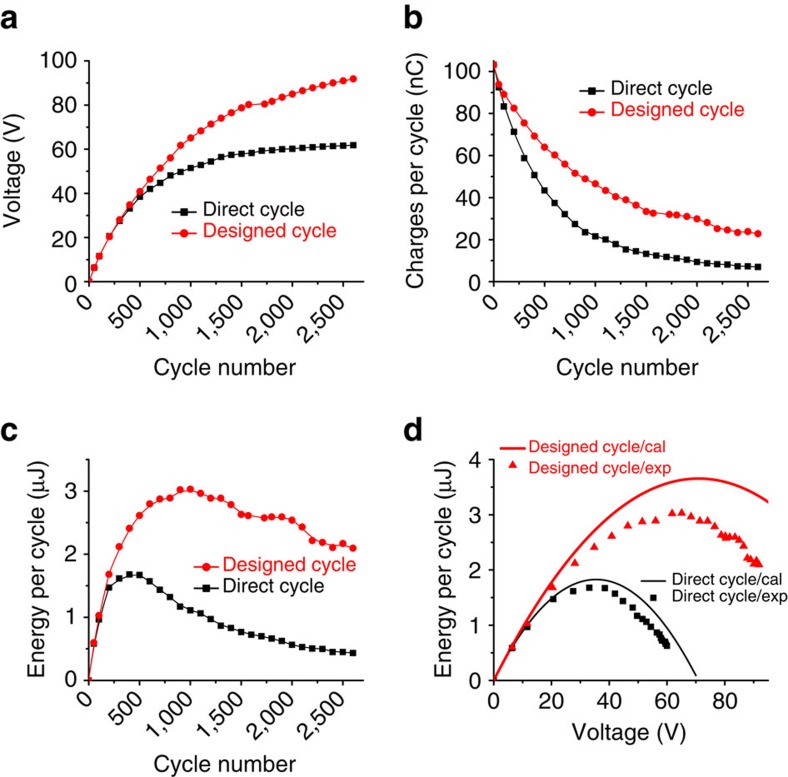
Quantitative comparisons between the charging cycles. (**a**) The charging voltage *V*_C_, (**b**) the amount of charge *Q*_C_ flowing to the capacitor per cycle and (**c**) the stored energy per cycle *E*_C_ changes versus the number of the charging cycles for both the direct and the designed charging cycles. (**d**) The experimental (dots) and calculated (lines) results (as calculated from equations (1) and (5)) of the stored energy per cycle *E*_C_ versus the charging voltage *V*_C_ for both the direct and the designed charging cycles.

To further reveal the advantages of the designed charging cycle in energy storage and verify our theoretical derivations above, quantitative comparisons between experimental results of the direct and the designed charging cycles are performed, as shown in Fig. 6. Here the plots for the capacitor are used since it can easily achieve different *V*_C_ during the charging process. Figure 6a–c show the changes of the charging voltage *V*_C_, the charge *Q*_C_ flowing to the capacitor per cycle and the stored energy per cycle *E*_C_ versus the number of the charging cycles, respectively. For both types of cycles, with the increase of the number of the charging cycles, *V*_C_ increases since more and more charges flow into the capacitor; *Q*_C_ decreases due to the increases of *V*_C_, as discussed in Supplementary Note 5. Compared with the direct charging cycle, *V*_C_ increases faster, *Q*_C_ decreases slower and hence *E*_C_ is significantly promoted in the designed charging cycle. The reason of slower decay of *Q*_C_ in the designed charging cycle is during the switch-on operations (statuses III to IV and VI to I), the charges are fully transferred to *Q*_SC,max_ or 0, so that in the next half-cycle there are more charges available to flow into the capacitor (as further discussed in Supplementary Note 5). We also notice that the higher saturation voltage *V*_Sat_ can be achieved in the designed charging cycle, since for the direct charging cycle there is no energy stored once *V*_C_ approaches *V*_Sat,direct_, as discussed in Supplementary Note 8. The curves of *E*_C_ versus *V*_C_ for both the direct and the designed charging cycles are also plotted in Fig. 6d for both experimental and calculated results (as calculated from equations (1) and (5)). From this we can conclude that, the designed charging cycle can supply a larger *E*_C_ under the same *V*_C_, and a higher *V*_Sat_ can be achieved.

### Application of the designed charging cycle

**Figure 7:**
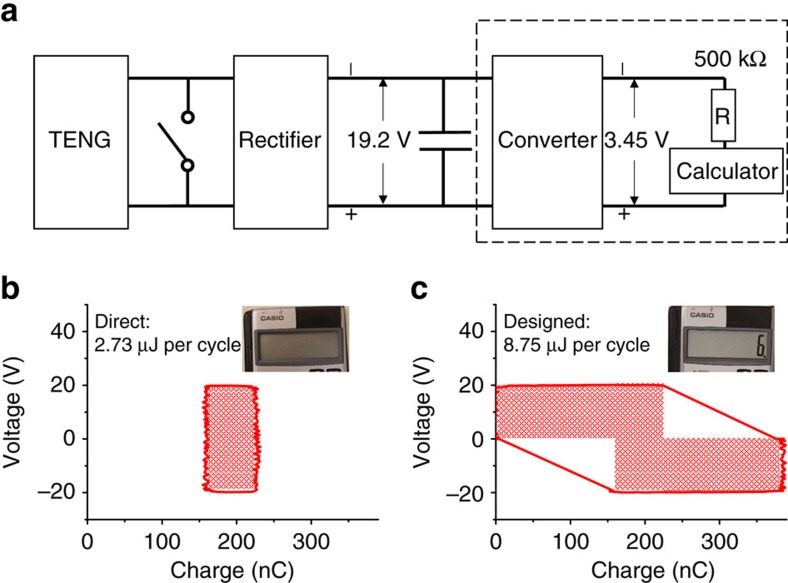
Demonstration of a calculator sustainably powered by the self-charging power system through the designed charging cycle. (**a**) Schematic diagram of the operation circuit; (**b**,**c**) the *V*–*Q* plots of the direct and the designed charging cycle for the TENG. The shaded areas represent the energy stored per cycle for both cycles. The power required by units in dashed frame in **a** is satisfied by that supplied by TENG through the designed cycle. The inset pictures show the calculator's display screen as charged by the direct and designed charging cycles, respectively.

To further demonstrate the promoted energy storage in the designed charging cycle, we use a TENG working in the designed charging cycle to power a commercial calculator (working voltage 1.5 V) in sustainable mode using the circuit in Fig. 7a. To provide a constant voltage to the calculator, the average input power from the TENG needs to equal to the power consumption in the output circuit^20^. The capacitor is charged to let *V*_C_=19.2 V initially. As indicated by the dashed frame in Fig. 7a, the output circuit, which is connected to the capacitor in parallel, includes a power converter (from Linear Technology) to regulate the voltage to 3.45 V, the calculator and a divider resistor of 500 kΩ to provide the calculator a voltage of ∼1.5 V. The measured current required to drive the output circuits is ∼1.5 μA (Supplementary Fig. 8), which indicates that an average output power of ∼28.8 μW needs to be provided by the TENG. If we set the working period as 0.3 s for the TENG, the required stored energy per cycle is ∼8.65 μJ. To achieve that, we fabricate a TENG that can generate ∼780 nC per cycle in maximum (*Q*_SC,max_≈390 nC). The measured *V*–*Q* plots of both the direct and the designed charging cycles at *V*_C_=19.2 V are shown in Fig. 7b,c. The measured *E*_C_ for the direct charging cycle is 2.73 μJ, which is much smaller than the required energy per cycle of 8.65 μJ. Consequently, the capacitor's energy operated in the direct charging cycle is consumed quickly, and the calculator turned off quickly, as shown in the inset of Fig. 7b. As a comparison, the measured *E*_C_ for the designed charging cycle is 8.75 μJ, which is larger than the required energy per cycle of 8.65 μJ. Therefore, as shown in the insets of Fig. 7c, with the same *V*_C_=19.2 V, the TENG operating in the designed charging cycle can drive the calculator sustainably. The calculator working in sustainable mode powered by the self-charging power system through the designed charging cycle is also shown in Supplementary Movie 1.

## Discussion

In summary, a rationally designed route for more effective energy storage of the random-pulsed output power generated by TENGs was developed theoretically and experimentally. The step-by-step charging processes of both the direct and the designed charging cycles were illustrated by *V*–*Q* plots, which revealed the advantages of the designed charging cycle in enhanced charging rate, improved energy-storage efficiency (up to 50%), and promoted saturation voltage (by at least a factor of two). As a proof of concept, we designed a TENG with motion-triggered switch to achieve the designed charging cycle. The results from both approaches were compared quantitatively, and the results confirmed the advantages of the designed charging cycle, as predicted by theoretical derivations. A commercial calculator was demonstrated to be sustainably powered by the TENG-based self-charging power system through the designed charging cycle. This designed charging cycle provides a route to enhance the energy harvesting and storage for TENGs as well as other nanogenerators, which represents a solid progress towards effectively utilizing ambient mechanical energy as a sustainable power source for electronics.

## Methods

### Fabrication and operation of the TENG

The static part of the TENG was fabricated by attaching Al foils on a 10.1 × 7 cm acrylic board to form two rectangular shape electrodes each with a size of 5 × 7 cm and the spacing of 1 mm. A 5 × 7-cm 50-μm FEP film attached along one side of a 5 × 15-cm acrylic board was used as the moving part. For the designed charging cycle, two Ti metal ribbons (1 cm in width for each, 5 cm in spacing) were attached on the other side of the acrylic board in parallel in the moving part, which were connected to two electrodes, respectively. Two Ti bars (7 cm in length) were also prepared. To operate the TENG in the designed charging cycle, the moving part was mounted on a linear motor and the static part and the two Ti bars were mounted on 3 three-dimensional stages, respectively, and the FEP surface and one Al electrode were placed face to face, as shown in Fig. 4d. The linear motor was controlled to move periodically between two Al electrodes with a displacement of 5.1 cm. For the direct charging cycle, the two Ti bars were removed. The TENG used to demonstrate the sustainable mode was fabricated in the same procedure with twice of all the sizes. The voltage and transferred charge were measured by two Keithley 6,514 system electrometers simultaneously. The measurement circuits for *V*–*Q* plots are illustrated in Supplementary Fig. 3.

### Fabrication of the lithium-ion battery

Lithium-ion rechargeable coin cell batteries were fabricated by using LiCoO_2_/carbon black/binder mixture on Al foil (1 cm in diameter) as the anode, polyethylene (PE) as separator (2 cm in diameter), the graphite/carbon black/binder mixture on Al foil (1.5 cm in diameter) as the cathode. The electrolyte (1 M LiPF_6_ in 1:1:1 ethylene carbonate/dimethyl carbonate/diethyl carbonate) was injected inside between the anode and cathode before the coin cell was pressed firmly. The charging-discharging curves of these batteries were tested as shown in Supplementary Fig. 9, which exhibits a plateau voltage of ∼3.8 V. In our experiments, each battery can supply voltage of ∼3–4 V.

## Additional information

**How to cite this article:** Zi, Y. *et al*. Effective energy storage from triboelectric nanogenerator. *Nat. Commun.* 7:10987 doi: 10.1038/ncomms10987 (2016).

## Supplementary Material

Supplementary Information Supplementary Figures 1-9, Supplementary Table 1, Supplementary Notes 1-8 and Supplementary References

Supplementary Movie 1 The calculator as powered by the designed charging cycle.

## Figures and Tables

**Table 1:** The definitions of important parameters of the TENG.

Symbol	Definitions
*Q*_SC,max_	The maximum short-circuit transferred charge
*V*_OC,max_	The maximum open-circuit voltage at *Q*=0
*V′*_max_	The maximum achievable absolute voltage at *Q*=*Q*_SC,max_
*E*_m_	The largest possible energy output per cycle
