# Supplementary material for: Effective energy storage from a triboelectric nanogenerator
Source: Nat Commun. 2016 Mar 11;7:10987. doi: 10.1038/ncomms10987 (PMC4793045; doi:10.1038/ncomms10987)
Supplement: Supplementary Information — Supplementary Figures 1-9, Supplementary Table 1, Supplementary Notes 1-8 and Supplementary References [file ncomms10987-s1.pdf]

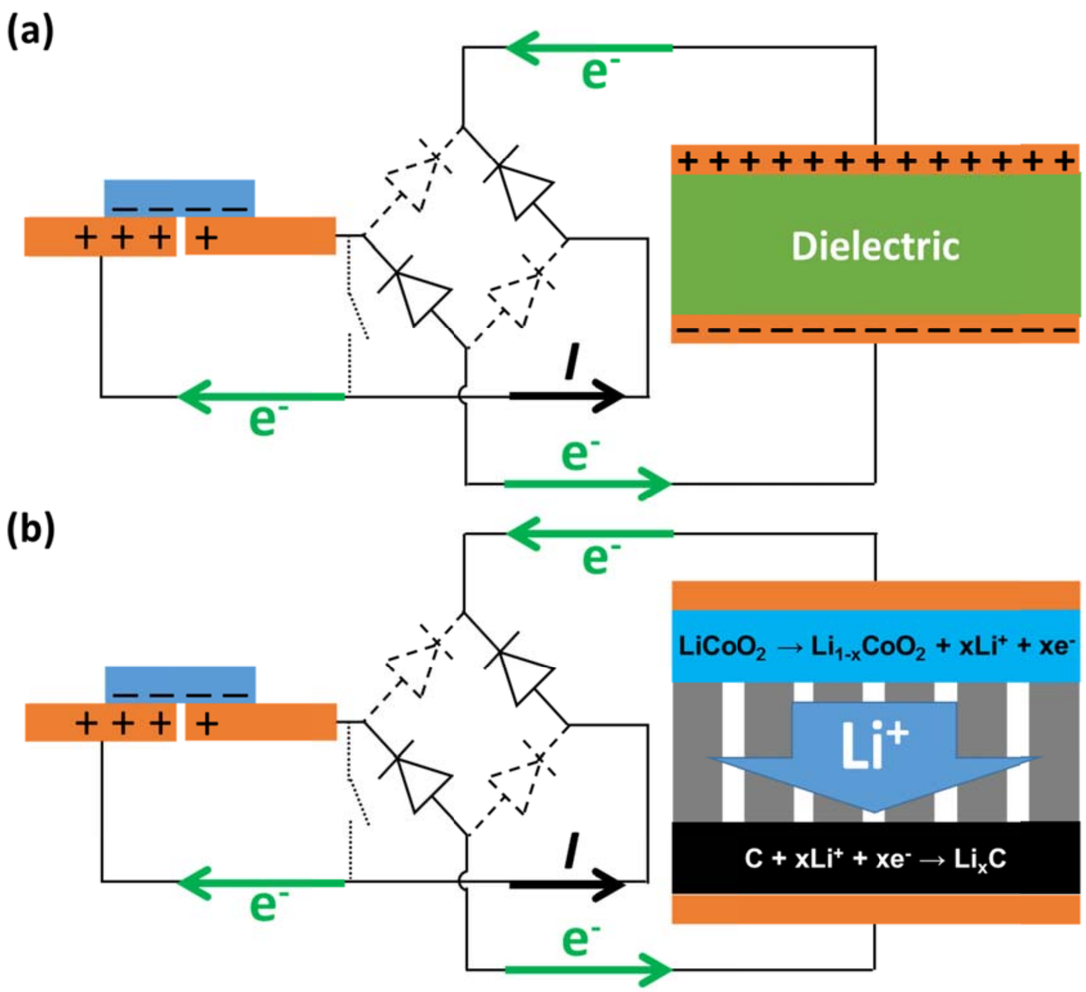

Supplementary Figure 1. The charging process of (a) a capacitor and (b) a fabricated lithium-ion battery. The switch exists for the designed charging cycle only.

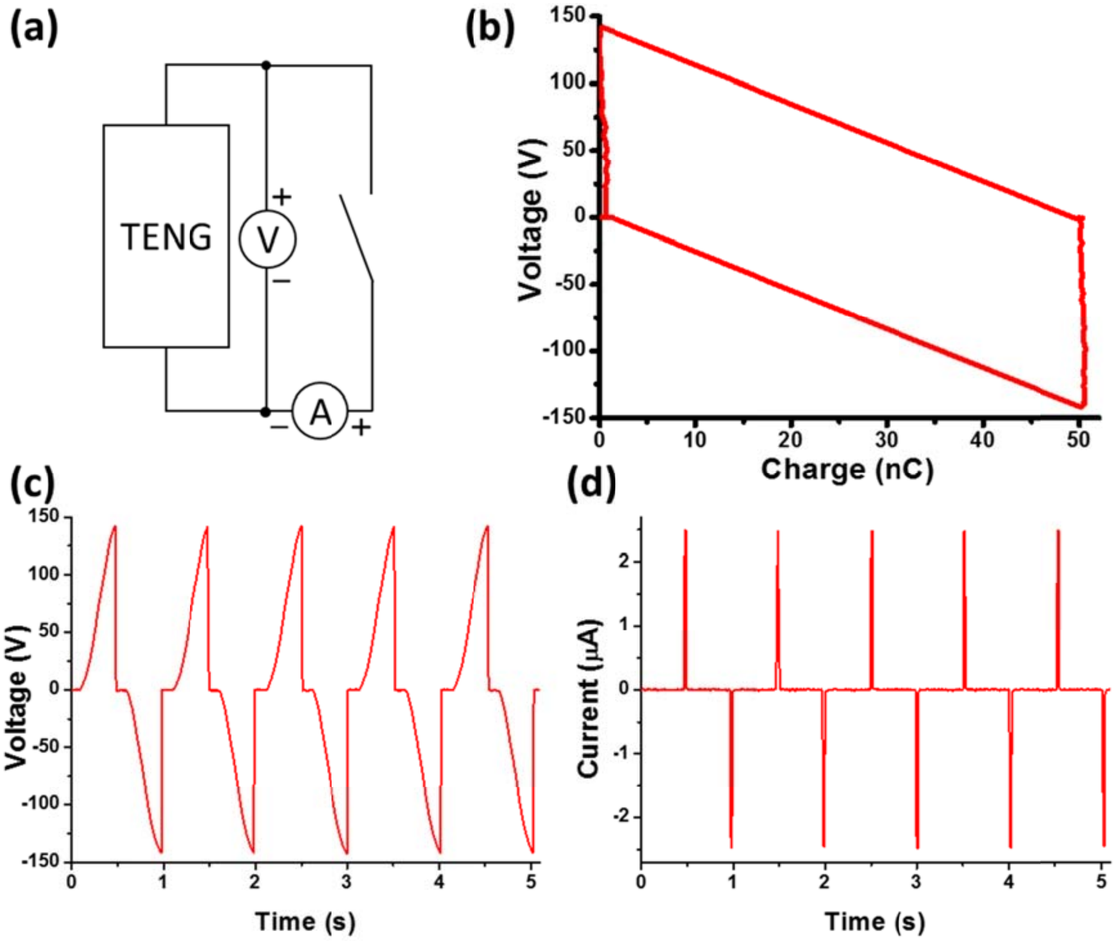

Supplementary Figure 2. (a) The measurement circuit diagram and (b) the cycle of maximized energy output (CMEO) with infinite load resistance of the fabricated TENG. (c) and (d) show the *V*-*t* and *I*-*t* plots of TENG under CMEO with infinite load resistance.

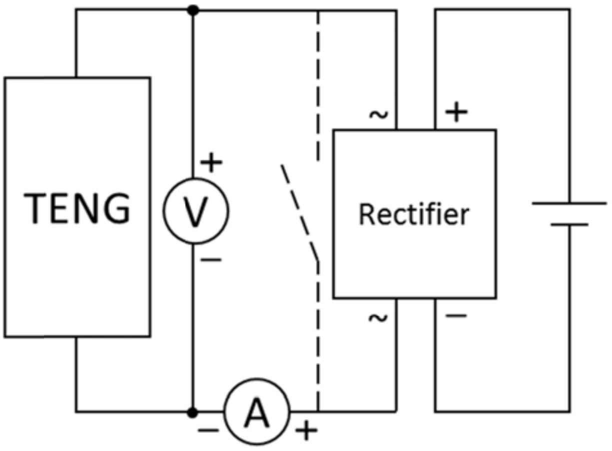

Supplementary Figure 3. The measurement circuit diagram for *V*-*Q* plots of the direct charging cycle and the designed charging cycle. The switch only exists for the designed charging cycle, and the battery can be replaced by the capacitor.

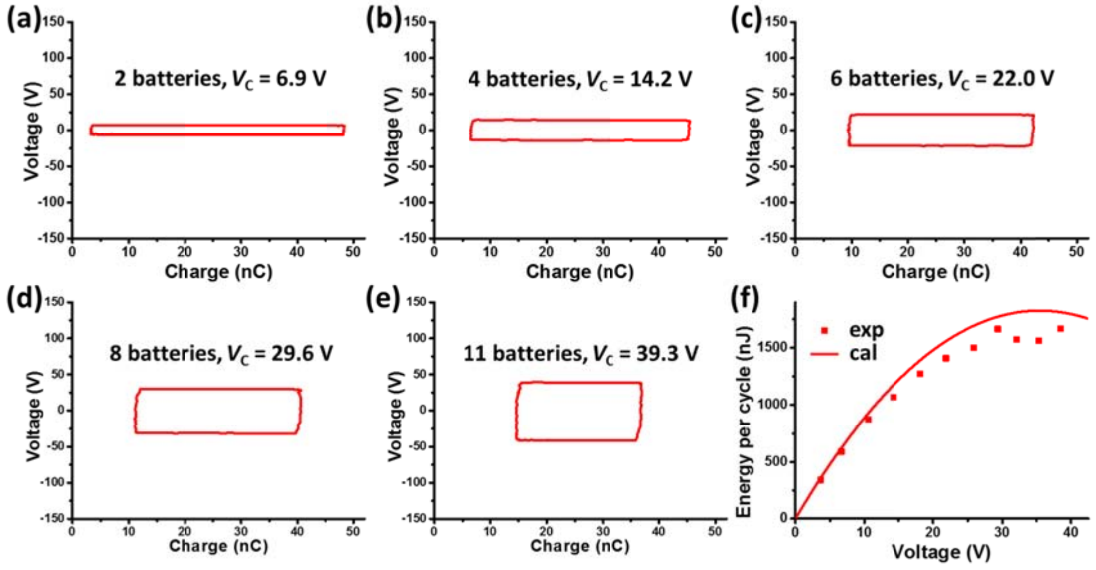

Supplementary Figure 4. (a)-(e) The measured direct charging cycles for TENG charging batteries in series. (f) The energy per cycle versus charging voltage as measured

by experiments (dots) and calculated by the Equation (1) in the article (line) in the direct charging cycles.

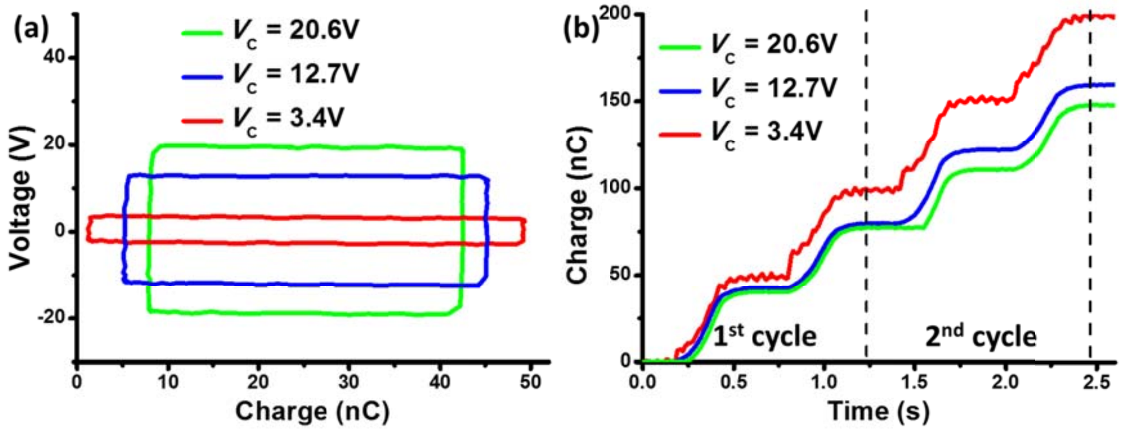

Supplementary Figure 5. The Q_C measured from (a) the *V-Q* curve in TENG side and (b) the *Q-t* curve in batteries/capacitor side. The results measured from the both methods are consistent to each other, as stated in Supplementary Note 6.

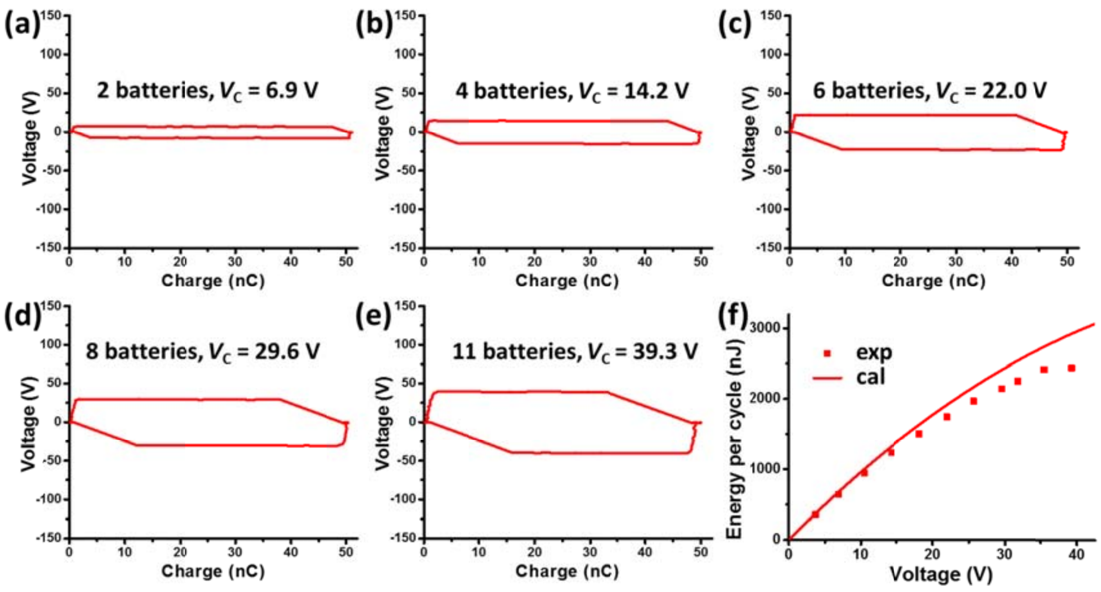

Supplementary Figure 6. (a)-(e) The measured designed charging cycles for TENG charging batteries in series. (f) The energy per cycle versus charging voltage as measured by experiments (dots) and calculated by Equation (5) in the article (line) in the designed charging cycles.

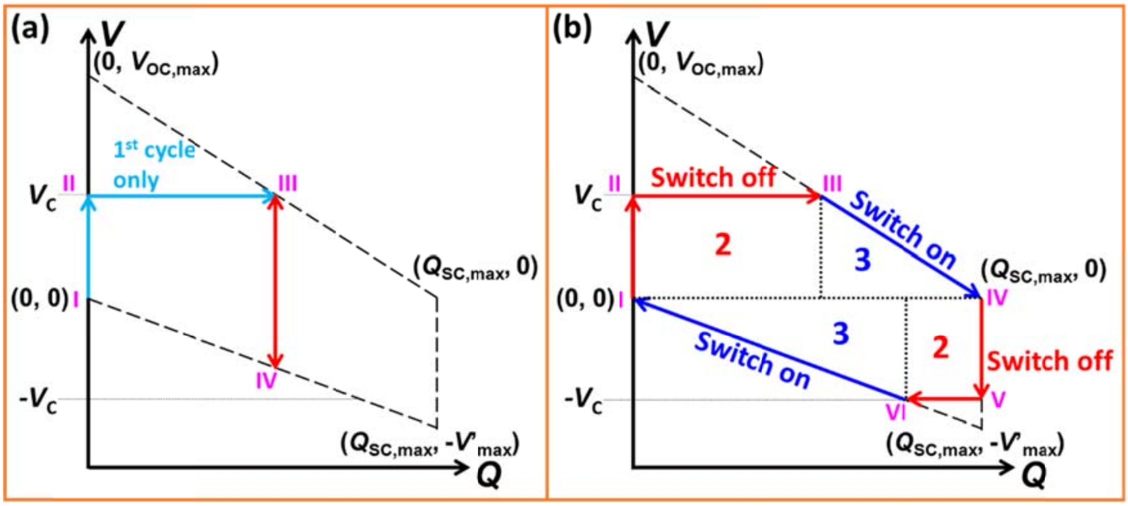

Supplementary Figure 7. The (a) direct and (b) designed charging cycles when V_C is larger than $V_{Sat,direct}$ but smaller than $V_{Sat,designed}$.

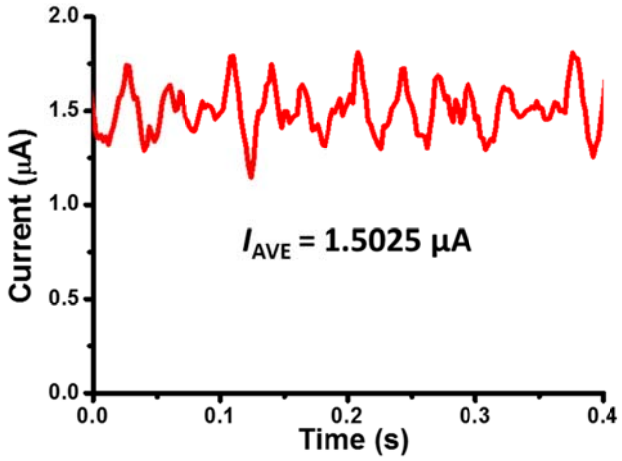

Supplementary Figure 8. The input current of the circuit in the dashed frame of Figure 7a as measured by using five batteries in series with total voltage of 19.2 V as V_C.

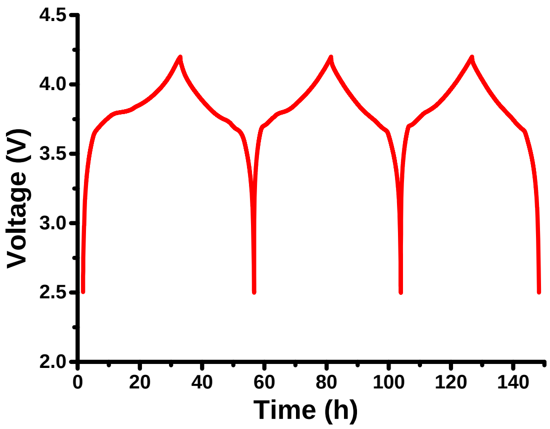

Supplementary Figure 9. The charging-discharging curve of one lithium-ion battery, which shows the plateau voltage of about 3.8 V.

Supplementary Table 1: Parameters about TENG energy storage

Symbol	Names and definitions
V_C	The charging voltage, the voltage of the energy storage unit
E_C	The stored energy per cycle (proportional to the average charging power)
$E_{C,direct}$	E_C for the direct charging cycle
$E_{C,designed}$	E_C for the designed charging cycle
$E_{C,direct,max}$	The maximum E_C for the direct charging cycle
$E_{C,designed,max}$	The maximum E_C for the designed charging cycle
Q_C	The amount of charge flowing to the energy storage unit (proportional to the average charging rate)
$Q_{C,direct}$	Q_C for the direct charging cycle
$Q_{C,designed}$	Q_C for the designed charging cycle
η	The energy storage efficiency, as the stored energy per cycle over E_m
η_{direct}	The maximum η of the direct charging cycle
$\eta_{designed}$	The maximum η of the designed charging cycle
V_{Sat}	The saturation voltage (the largest possible charging voltage)
$V_{Sat,direct}$	V_{Sat} of the direct charging cycle
$V_{Sat,designed}$	V_{Sat} of the designed charging cycle

Supplementary Note 1: The equations for boundaries of CMEO with infinite load *R*: As demonstrated previously[1], $Q = 0$ and $Q = Q_{\mathrm{SC,max}}$ are the two boundaries which are parallel to $V-$axis. The other two boundaries are:

$$\frac{V}{V_{\mathrm{OC,max}}}+\frac{Q}{Q_{\mathrm{SC,max}}}=1\ (\text{for } x=x_{\mathrm{max}}) \tag{1}$$

$$\frac{V}{V'_{\mathrm{max}}}+\frac{Q}{Q_{\mathrm{SC,max}}}=0\ (\text{for } x=0) \tag{2}$$

The equations (1) and (2) can be used to calculate coordinates of important points in the charging cycles.

Supplementary Note 2: The change of V_{C} during one charging cycle: For the batteries, we can use the region close to the plateau voltage, then V_{C} will be nearly constant during charging process. For capacitors, most of load capacitors used for charging have a large capacitance C_{L}. In fact as reported, to achieve efficient charging process, the optimum value of C_{L} should be much larger than the largest value of $C(x)$[2]. The capacitance between two electrodes of TENG can be written as a variable $C(x)$ related to the displacement x, and it is usually very small. Therefore:

$$\frac{Q_{\mathrm{SC,max}}}{\Delta V_{\mathrm{C}}}>\frac{\Delta Q}{\Delta V_{\mathrm{C}}}=C_{\mathrm{L}}>>C(x)_{\mathrm{max}}=\frac{Q_{\mathrm{SC,max}}}{\min\{V_{\mathrm{OC,max}},V'_{\mathrm{max}}\}} \tag{3}$$

Here, ΔQ and ΔV_{C} represent the amount of charges flowing to the capacitor and the variation of V_{C} in the capacitor during a half charging cycle. Then we can deduct that $\min\{V_{\mathrm{OC,max}}, V'_{\mathrm{max}}\} >> \Delta V_{\mathrm{C}}$, which means we can assume that charging voltage V_{C} does not change significantly during one charging cycle.

Supplementary Note 3: The process of TENG charging the capacitor/battery: During the charging process of a capacitor, the electrons from one electrode of the TENG are driven to the negative electrode of the capacitor. Consequently, the additional positive charge is induced in the positive electrode by the electrostatic induction, and the additional electrons are released from the positive electrode in the capacitor, and driven to the other electrode of the TENG.

During the charging process of one fabricated lithium-ion battery or several batteries in series, the electrons from one electrode of the TENG are driven to the cathode of the first battery, to facilitate the following reaction in the cathode:

$$C + xLi^{+} + xe^{-} \rightarrow Li_xC \quad (4)$$

With the consumption of the lithium ions, the ones in the anode immigrate to the cathode to balance the concentration. Therefore, the following reaction in the anode is promoted to release more lithium ions:

$$LiCoO_2 \rightarrow Li_{1-x}CoO_2 + xLi^{+} + xe^{-} \quad (5)$$

If there are batteries in series, the additional electrons are driven to the cathode of the next battery. The process and reactions in the other batteries are as same as the ones in the first battery. Eventually the additional electrons from the anode of the last battery flow to the other electrode of the TENG.

The process and reactions stated are schematically shown in Supplementary Figure 1.

Supplementary Note 4: Calculation of coordinates in the charging cycles: For the direct charging cycle, apparently status I is (0, 0), and II is (0, V_C). The status III is with $x = x_{max}$, which should satisfy Supplementary Equation (1). And since $V = V_C$, then $Q =$

$Q_{SC,max}$ (1 - $V_C/V_{OC,max}$). So status III is ($Q_{SC,max}$ (1 - $V_C/V_{OC,max}$), V_C). From status III to status IV, all of the rectifiers are off, so there is no charge transfers (Q is kept). Then status IV is ($Q_{SC,max}$ (1 - $V_C/V_{OC,max}$), -V_C). The status V is with x = 0, which should satisfy Supplementary Equation (2). And since V = -V_C, then Q = $Q_{SC,max}$ V_C/V'_{max}. So status V is ($Q_{SC,max}$ V_C/V'_{max}, - V_C). From status V to VI, all of the rectifiers are off, then Q is kept and status VI is ($Q_{SC,max}$ V_C/V'_{max}, V_C).

Similarly, for the designed charging cycle, statuses I to III are calculated as (0, 0), (0, V_C) and ($Q_{SC,max}$ (1 - $V_C/V_{OC,max}$), V_C), respectively. In status IV which still satisfies Supplementary Equation (1), the switch is on to make V = 0. Then status IV is calculated as ($Q_{SC,max}$, 0). The status V is given by Q = $Q_{SC,max}$ since all of the rectifiers are off. So status V is ($Q_{SC,max}$, - V_C). The status VI which is with x = 0 is as same as status V in the direct charging cycle.

Supplementary Note 5: The decrease of Q_C as increase of V_C in the charging cycles:

The calculation method of coordinates has been stated in Supplementary Note 4. Then we can extract Q_C in both cases as:

$$Q_{C,direct} = 2[Q_{SC,max}(1-V_C/V_{OC,max}) - Q_{SC,max}V_C/V'_{max}] = 2Q_{SC,max}(1-V_C/V_{OC,max} - V_C/V'_{max}) \quad (6)$$

$$Q_{C,designed} = Q_{SC,max}(1-V_C/V_{OC,max}) + Q_{SC,max} - 0 - Q_{SC,max}V_C/V'_{max}$$
$$= Q_{SC,max}(2-V_C/V_{OC,max} - V_C/V'_{max}) \quad (7)$$

As we can see, both Q_C decrease with increase of V_C. The reason is discussed below:

When x is approaching x_{max} from 0, a certain charge of $Q_{SC,max}V_C/V_{OC,max}$ should stay in the 1st electrode (the electrode overlapped by the dielectric when x = 0 in SFT mode) to

the maintained voltage of V_C; similarly, when x is approaching 0 from x_{max}, a certain charge of $Q_{SC,max} V_C / V'_{max}$ should stay in the 2nd electrode (the electrode overlapped by the dielectric when $x = x_{max}$ in SFT mode) due to the maintained voltage of - V_C. And these amounts of charges are both proportional to V_C. Even through in the designed charging cycle, the switch is turned on to make charges fully transferred, these additionally transferred charges are through the switch other than the energy storage unit. Thus, at increased V_C, as limited by these parts of charges, Q_C for both charging cycles decrease.

The difference between Q_C for both charging cycles is:

$$Q_{C,designed} - Q_{C,direct} = Q_{SC,max}(V_C / V_{OC,max} + V_C / V'_{max}) > 0 \quad (8)$$

So in the designed charging cycle, Q_C will decrease slower than the direct charging cycle. The fundamental reason of that is stated as below:

During switch-on operation from statuses III to IV, the charges were fully transferred to $Q_{SC,max}$, so that in the next half-cycle, the charges available for flowing to the energy storage unit change from $Q_{SC,max}(1 - V_C / V_{OC,max} - V_C / V'_{max})$ (differences in Q between statues IV and V in the direct charging cycle) to $Q_{SC,max}(1 - V_C / V'_{max})$ (differences in Q between statues V and VI in the designed charging cycle);

Similarly, during switch-on operation from statuses VI to I, the charges were fully transferred to 0, so that in the next half-cycle, the charges available for flowing to the energy storage unit change from $Q_{SC,max}(1 - V_C / V_{OC,max} - V_C / V'_{max})$ (differences in Q between statues III and VI in the direct charging cycle) to $Q_{SC,max}(1 - V_C / V_{OC,max})$ (differences in Q between statues III and II in the designed charging cycle);

Supplementary Note 6: Q_C for both the direct and designed charging cycles: Theoretically, only when the voltage V between the electrodes of TENG achieves the charging voltage V_C, the charging process of the batteries/capacitors can proceed. In both the shapes of the direct and the designed charging cycles, the sides parallel to the Q axis means V is kept at a constant voltage as $\pm V_C$, therefore, Q_C is the total length of these sides. To double confirm that, we measured Q_C directly by connecting an Ammeter in series with the batteries/capacitor in the circuit for the direct charging cycles, as shown in Supplementary Figure 5. Q_C measured here are 99.8 nC for V_C = 3.4 V, 79.6 nC for V_C = 12.7 V and 73.9 nC for V_C = 20.6 V. These measured Q_C are consistent with the measured total length of the sides parallel to the Q-axis in the corresponding V-Q curves of the direct charging cycles, which are 96.3 nC for V_C = 3.4 V, 80.5 nC for V_C = 12.7 V, and 71.3 nC for V_C = 20.6 V.

Supplementary Note 7: The symmetric roles of $V_{OC,max}$ and V'_{max}: $V_{OC,max}$ and V'_{max} are symmetric since they are transferrable to each other while a different coordinate was used. If we transfer the coordinate as $(Q', V') = (Q_{SC,max} - Q, -V)$, and then, the $(0, V_{OC,max})$ and $(Q_{SC,max}, -V'_{max})$ in the old coordinate will become $(Q_{SC,max}, -V_{OC,max})$ and $(0, V'_{max})$ in the new coordinate as the two points with the largest open-circuit negative and positive voltages. Thus, $V_{OC,max}$ and V'_{max} are transferred to each other in this new coordinate.

Supplementary Note 8: The charging cycles when V_C is larger than $V_{Sat,direct}$: The calculation method of coordinates has been stated in Supplementary Note 4.

If $V_C \geq V_{OC,max}$ (we can assume $V_{OC,max} \geq V'_{max}$ as we stated in Supplementary Note 7), the rectifiers cannot be opened, then no energy harvesting in both the direct and designed cycles;

If $V_{Sat,direct} = \frac{V_{OC,max}V'_{max}}{V_{OC,max}+V'_{max}} \leq V_C < V_{OC,max}$ (Please check Equation (4) in the manuscript for $V_{Sat,direct}$), for the direct charging cycle, the process from status I to status III (the 1st half cycle) is as same as described in Figure 1. The V-Q plot corresponding to this process is plotted in Supplementary Figure 7a. The corresponding status III is at ($Q_{SC,max}$(1-$V_C/V_{OC,max}$), V_C). And then when x changes from x_{max} to 0, the rectifiers are all turned off and Q is kept at $Q_{SC,max}$(1-$V_C/V_{OC,max}$). If we assume this open-circuit condition is kept until x = 0, the voltage of status IV in Supplementary Figure 7a is (satisfying Supplementary Equation (2)):

$$V = -V'_{max}\frac{Q}{Q_{SC,max}} = -V'_{max}(1-\frac{V_C}{V_{OC,max}}) \geq -V'_{max}(1-\frac{V_{Sat,direct}}{V_{OC,max}}) = -V_{Sat,direct} \geq -V_C \qquad (9)$$

From status III to IV (except right at status III or IV), $|V| < V_C$, so the rectifiers cannot be opened again. And in following cycles, TENG oscillates between status III and status IV. Consequently, $|V| < V_C$ is always valid for $0 < x < x_{max}$, rectifiers cannot be opened, and there is no energy stored. Only when $x = 0$ or x_{max}, $|V|$ might equal to V_C, but since the displacement x cannot change further (as fixed in the range of 0 to x_{max}), no charge can be pushed into the energy storage unit. In summary, in this case, the energy can only be stored in the 1st half cycle, and there is no energy storage during steady-state of the direct charging cycle. So in the continuous direct charging cycle for a capacitor, when V_C is

approaching $V_{Sat,direct}$, less and less energy is stored in the capacitor; and $V_C > V_{Sat,direct}$ is not accessible since there is no energy stored once $V_C = V_{Sat,direct}$.

For the designed charging cycle, as shown in Supplementary Figure 7b, the process is still cycled from status I to VI, as same as that in the manuscript. The reason is that after statuses III and VI, the switch is turned on to make $V = 0$ (statuses IV and I), so that $|V|$ can achieve $|V_C|$ much easier in the following steps when all of the rectifiers are all off (status IV to V and status I to II). However, in this case the encircled area only contains area 2 which can only be stored by the designed charging cycle, and area 3 which is released through the switch. The area 1 that can be stored by both the direct and designed charging cycles as described in Figure 3 is missing due to the reason as stated above. So in this designed charging cycle, the charging process can continue after V_C surpass $V_{Sat,direct}$, until V_C is approaching max{$V_{OC,max}$, V'_{max}}.

Supplementary References:

1 Zi, Y. *et al.* Standards and Figure-of-Merits for Quantifying the Performance of Triboelectric Nanogenerators. *Nature Communications* **6**:8376 (2015).

2 Niu, S. *et al.* Optimization of Triboelectric Nanogenerator Charging Systems for Efficient Energy Harvesting and Storage. *Electron Devices, IEEE Transactions on* **62**, 641-647 (2015).
